# Plasma Extracellular Vesicle Size and Concentration Are Altered in Alzheimer’s Disease, Dementia With Lewy Bodies, and Frontotemporal Dementia

**DOI:** 10.3389/fcell.2021.667369

**Published:** 2021-05-11

**Authors:** Antonio Longobardi, Luisa Benussi, Roland Nicsanu, Sonia Bellini, Clarissa Ferrari, Claudia Saraceno, Roberta Zanardini, Marcella Catania, Giuseppe Di Fede, Rosanna Squitti, Giuliano Binetti, Roberta Ghidoni

**Affiliations:** ^1^Molecular Markers Laboratory, IRCCS Istituto Centro San Giovanni di Dio Fatebenefratelli, Brescia, Italy; ^2^Service of Statistics, IRCCS Istituto Centro San Giovanni di Dio Fatebenefratelli, Brescia, Italy; ^3^Neurology 5/Neuropathology Unit, Fondazione IRCCS Istituto Neurologico Carlo Besta, Milan, Italy; ^4^MAC-Memory Clinic, IRCCS Istituto Centro San Giovanni di Dio Fatebenefratelli, Brescia, Italy

**Keywords:** Alzheimer’s disease, dementia with Lewy bodies, frontotemporal dementia, extracellular vesicles, nanoparticle tracking analysis, biomarkers, plasma

## Abstract

Alzheimer’s disease (AD), frontotemporal dementia (FTD), and dementia with Lewy bodies (DLB) are the three major neurodegenerative dementias. In this study, we provide evidence that an alteration in extracellular vesicles (EVs) release is common across the three most common neurodegenerative dementias, AD, DLB, and FTD. Specifically, we analyzed plasma EVs in three groups of patients affected by AD, DLB, and FTD, and we found a significant reduction in EVs concentration and larger EVs size in all patient groups. We then investigated whether the loss of neurotrophic factors is also a common pathogenic mechanism among FTD, DLB, and AD, and if levels of neurotrophic factors might affect EVs release. Plasma levels of progranulin and cystatin C (CysC) were partially altered; however, taking together all variables significantly associated with the diagnostic groups only EVs size and concentration were able to distinguish patients from controls. The diagnostic performance of these two EVs parameters together (ratio) was high, with a sensitivity of 83.3% and a specificity of 86.7%, able to distinguish patients from controls but not to differentiate the different forms of dementias. Among the candidate neurotrophic factors, only CysC levels were associated with EVs concentration. Our study suggests that an alteration in the intercellular communication mediated by EVs might be a common molecular pathway underlying neurodegenerative dementias. The identification of shared disease mechanisms is of pivotal importance to develop treatments to delay disease progression. To this aim, further studies investigating plasma EVs size and concentration as early biomarkers of dementia are required.

## Introduction

Alzheimer’s disease (AD), frontotemporal dementia (FTD), and dementia with Lewy bodies (DLB) are the three major neurodegenerative dementias. Brain abnormal protein accumulation and inclusions characterize all these neurodegenerative diseases (Jellinger, 2008; Soto and Estrada, 2008): AD is characterized by deposition of beta-amyloid peptides (Aβ) in amyloid plaques and deposition of phosphorylated tau protein in neurofibrillary tangles (Hardy and Higgins, 1992; Brion, 1998); DLB is characterized by α-synuclein inclusions in neurons, neurites, glia, and presynaptic terminals (Beyer et al., 2009; Donaghy and McKeith, 2014); FTD includes different pathological subtypes, characterized by tau, ubiquitin, Fused-in-Sarcoma (FUS), and TAR DNA-binding protein 43 (TDP-43)-positive inclusions (Mackenzie and Neumann, 2016; Neumann and Mackenzie, 2019). It is now clear that the protein aggregates spread from neuron to neuron contributing to the progression of the disease (Goedert, 2015). Exosomes, a specific subtype of extracellular vesicles (EVs) of endosomal origin, are capable of transferring biomolecules between cells without direct cell-to-cell contact (Raposo and Stoorvogel, 2013). Thus, EVs, and specifically exosomes, have been suggested as potential carriers of misfolded toxic proteins, such as Aβ peptide and tau in AD (Saman et al., 2012; Rajendran et al., 2014) and α-synuclein in Parkinson disease (PD)/DLB (Emmanouilidou et al., 2010; Alvarez-Erviti et al., 2011). Regarding AD, the discovery that Aβ precursors and members of the secretase complex are secreted within exosomes grew interest in EVs in the last decade, demonstrating that they are actively involved in Aβ peptide generation and plaque formation (Ghidoni et al., 2011). Since EVs can cross the blood–brain barrier and reach the blood, it was possible to identify several potential biomarkers in exosomes isolated from blood: increased levels of *t*-tau, *p*-tau, and Aβ42 in plasma/serum neurally derived blood exosomes were demonstrated to be an early marker for AD and cognitive decline progression (Fiandaca et al., 2015). Conversely, in blood-derived exosomes several synaptic proteins as well as survival proteins were described to be reduced in AD and FTD (Goetzl et al., 2015; Goetzl et al., 2016; Goetzl et al., 2018). In addition, we demonstrated that CysC, a trophic protein targeted to the classical secretory pathway, is secreted by mouse primary neurons in association with exosomes and the overexpression of familial AD-associated presenilin 2 mutations (PS2 M239I and PS2 T122R) resulted in a loss of exosomal CysC and of Aβ precursor protein (APP) metabolites within exosomes (Ghidoni et al., 2011). FTD-causing progranulin null mutations cause a loss of progranulin (PGRN), a neurotrophic factor (Ghidoni et al., 2008b); we demonstrated that also PGRN, a protein targeted to the classical secretory pathway, is secreted in association with exosomes by human primary fibroblasts and that null mutations in the GRN gene strongly reduce the number of released exosomes and alter their composition (Benussi et al., 2016). Brain-derived neurotrophic factor (BDNF), a key regulator of neuronal survival, was suggested to play an important role in the pathophysiology of neurodegenerative diseases including AD, FTD, and DLB (Ventriglia et al., 2013; Mitre et al., 2017). Moreover, some studies suggested the efficacy of neurotrophic factors (NTF) for the treatment of PD and DLB (Ramaswamy et al., 2009; Goldberg et al., 2015). A systemic administration of glial-derived neurotrophic factor (GDNF)-expressing macrophages significantly ameliorated neurodegeneration and neuroinflammation in PD mice, and one of the suggested mechanisms for this effect was the release of exosomes containing the GDNF, followed by the efficient GDNF transfer to target neurons (Zhao et al., 2014).

During progressive neurodegeneration and neuronal loss, exosomes could become the key player for neuronal communication and the crossroads of proteins: accordingly, survival of neurons could be easily affected by factors modulating exosome release and/or composition (Ghidoni et al., 2008a). We hypothesize that an alteration in exosome release and or composition might be a common pathological mechanism across the three major neurodegenerative dementias influencing the fate of disease-related proteins across dementias. Loss of NTF might be one of the determinants affecting exosome release.

To test our hypothesis, herein we analyzed human plasma EVs and NTF (CysC, PGRN, BDNF, and GDNF) in dementia patients (AD, DLB, and FTD diagnoses), investigating also the link between NTF and EVs.

## Materials and Methods

### Participants

Human plasma samples from *n* = 30 AD, *n* = 30 DLB, *n* = 30 FTD sporadic patients and from *n* = 30 elderly subjects with normal cognitive function, as control group (CTRL), were analyzed. Patients were enrolled at the MAC Memory Clinic IRCCS Fatebenefratelli, Brescia, and at the Neurology 5/Neuropathology Unit, IRCCS Besta, Milan. Clinical diagnosis for probable AD, DLB, and FTD was made according to international guidelines (McKhann et al., 1984; McKeith et al., 1996; Neary et al., 1998; McKhann et al., 2011; Rascovsky et al., 2011): patient diagnosis was derived by neurologists and neuropsychologists, who performed extensive behavioral, neuropsychological, and neuroimaging assessments. Differential diagnosis was supported by CSF analysis and 18-FDG PET/dopamine-transporter scan, when available. Clinical and demographic characteristics are shown in Table 1. Patients provided written informed consent. The study protocol was approved by the local ethics committee (Prot. N. 111/2017).

**TABLE 1:** Clinical, demographic, and biological variables of patients and controls.

	CTRL (*n* = 30)	AD (*n* = 30)	DLB (*n* = 30)	FTD (*n* = 30)	*p*-value
Gender (M:F)	8:22	9:21	17:13	13:17	0.069^†^
Age, years	65.8 ± 10.5	69.0 ± 7.8	**75.4 ± 6.9**	65.8 ± 8.5	<0.001^∗^
Disease onset, years	–	65.2 ± 8.2	**71.0 ± 7.7**	62.8 ± 6.9	0.002^∗^
Education, years	**11.9 ± 3.2**	7.2 ± 3.9	7.9 ± 3.7	7.4 ± 4.2	<0.001^∗∗^
MMSE	**28.9 ± 1.2**	19.4 ± 4.9	20.7 ± 5.8	19.0 ± 6.7	<0.001^∗∗^
EVs concentration, EVs/ml	**2.49 × 10^11^ ± 1.18 × 10^11^**	1.47 × 10^11^ ± 6.08 × 10^10^	1.26 × 10^11^ ± 5.10 × 10^10^	1.39 × 10^11^ ± 6.14 × 10^10^	<0.001^∗^
EVs size, nm	**113.2 ± 13.2**	133.8 ± 17.1	127.2 ± 12.0	130.2 ± 18.8	<0.001^∗^
BDNF, pg/ml	1,142.0 ± 1,798.4	631.6 ± 545.7	833.6 ± 740.1	765.3 ± 771.6	0.840^∗∗^
PGRN, ng/ml	38.8 ± 9.3	32.7 ± 7.3	37.9 ± 15.0	**31.1 ± 11.6**	0.003^∗∗^
CysC, ng/ml	881.8 ± 282.1	949.3 ± 247.3	**1,064.0 ± 270.2**	1,001.0 ± 301.2	0.043^∗∗^

All continuous variables were analyzed with the Kolmogorov–Smirnov test to evaluate normality. CTRL, controls; AD, Alzheimer’s disease patients; DLB, dementia with Lewy bodies patients; FTD, frontotemporal dementia patients; MMSE, Mini-Mental State Examination. ^†^Chi-square test; *One-way ANOVA test; **Kruskal–Wallis test. In bold is reported the group which differs from others (post hoc tests). Means ± standard deviation.

### Evs Isolation

EVs isolation was performed with Total Exosome Isolation Kit from plasma (Invitrogen^TM^, California, United States) following the manufacturer’s protocol. Briefly, 125 μl of plasma was centrifuged at 2,000 × g for 20′ and then at 10,000 × g for 20′; supernatants were then transferred into new tubes, mixed with 62.5 μl (0.5 × plasma volume) of 0.2 μm filtered 1× phosphate-buffered saline (PBS), and then incubated with 37.5 μl (0.2 × total volume) of Exosome Precipitation Reagent for 10′ at room temperature; after incubation, samples were then centrifuged at 10,000 × g for 5′. EVs pellets were resuspended in 100 μl of 0.2 μm filtered 1× PBS and stored at + 4°C or −20°C until nanoparticle tracking analysis (NTA). As a negative control, an aliquot of 125 μl of 1× PBS was processed as described above.

### Nanoparticle Tracking Analysis (NTA)

Suspension containing EVs from all four groups were analyzed with the Nano-Sight NS300 Instrument (Malvern, Worcestershire, United Kingdom). Samples were diluted with 0.2 μm filtered 1× PBS in order to obtain an optimal range of 20–150 particles/frame. For each sample, 5 videos of 60″ duration were recorded and data were processed using NanoSight NTA Software 3.2. Post-acquisition settings were kept constant between samples. Data obtained were: particle concentration (particles/ml), average size (nm), and particle size distribution (D10, D50, D90: particle size values indicating that, respectively, 10, 50, and 90% of the distribution is below this value). Raw concentration data (particles/ml) obtained from NTA were normalized to obtain EVs concentrations in the human plasma sample.

### Biochemical Analyses

GDNF, BDNF, and PGRN plasma concentrations were measured with Human Premixed Multiplex—Magnetic Luminex^®^ Assays (R&D Systems^®^, Minneapolis, United States) following the manufacturer’s protocol. CysC plasma concentration was measured with Human Cystatin C Quantikine^®^ ELISA kit (R&D Systems^®^, Minneapolis, United States) following the manufacturer’s protocol. All analyses were performed in duplicate. Measurements were carried out at the same study site on consecutive days, and researchers were unaware of whether the sample belonged to cases or to controls.

### Statistical Analysis

Normality assumption of continuous variables was evaluated with Kolmogorov–Smirnov. One-way ANOVA, with Bonferroni *post hoc* tests, was used for the comparison across the four subject groups of the normally distributed continuous variables. Kruskal–Wallis test with Dunn’s *post hoc* tests was used for the group comparisons of non-normally distributed variables. Chi-square test was used to assess the association between demographic characteristic (categorical variables) of subjects with the four groups. A classification tree (CT) (James et al., 2015) was applied to detect the best (in terms of classification performance) predictors for discriminating controls versus patients’ group. CT method was carried out on the diagnostic group as a categorical dependent variable depending on categorical and/or quantitative covariates. The output of the CT is given by different classification pathways (defined by estimated covariate cut offs), and for each of them, the probability of the most likely diagnostic group is provided. In order to take into consideration all possible socio-demographic and clinical confounders, in CT all variables which differ among groups were included. Diagnostic performance of EVs concentration and EVs size in discriminating across the four groups was assessed by areas under the curve (AUC) obtained by receiver operating characteristic (ROC). Comparison of the four AUC was assessed by DeLong test. Regression analyses were performed on EVs concentration and size (as dependent variables, respectively) and NTF, group, and their interaction as independent variables. All analyses were performed by SPSS software and significance set at 0.05.

## Results

EVs isolated from plasma samples of dementia patients (AD *n* = 30, DLB *n* = 30, and FTD *n* = 30) and cognitively healthy controls (CTRL *n* = 30) were analyzed (Table 1). NTA revealed EVs concentrations ranging from 4.16 × 10^10^ to 5.04 × 10^11^ EVs/ml and particle size from 81.5 to 177.7 nm (D50: 69.7–152.4 nm; D90: 115.3–314.9 nm). Thus, the EVs preparations were enriched in exosomes. The negative control was below the detection limit, thus not interfering with the sample analysis (data not shown).

**FIGURE 1:**
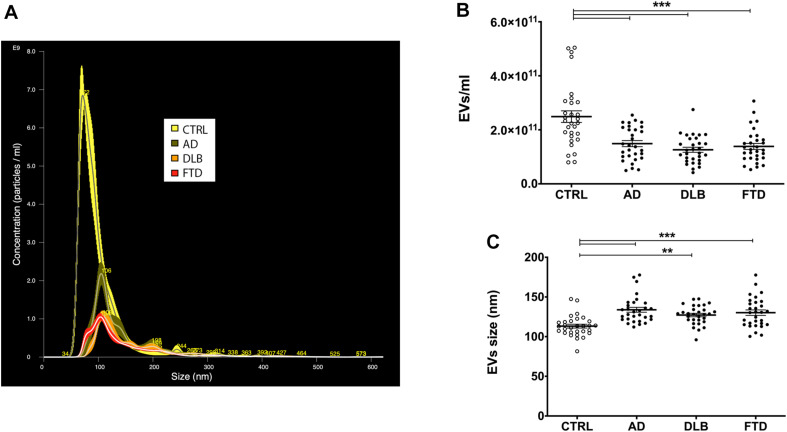
EVs concentration and size in patient and control groups. **(A)** Representative spectra from NTA of CTRL (yellow), AD (green), DLB (orange), and FTD (red) plasma EVs. **(B)** Quantification of EVs concentration with NTA in CTRL, AD, DLB, and FTD plasma samples. A statistically significant decrease in EVs concentration was observed in the three pathological groups compared to controls. **(C)** Representation of EVs size measured with NTA in CTRL, AD, DLB, and FTD plasma samples. An increase in size was observed in AD, DLB, and FTD compared to CTRL samples. Average ± SEM; **p < 0.01, ***p < 0.001, one-way ANOVA with Bonferroni’s post test.

NTA showed a significant decrease in plasma EVs concentration in AD, DLB, and FTD samples compared to CTRL samples (Table 1 and Figures 1A,B) (*p* < 0.001, one-way ANOVA test with Bonferroni’s post test, CTRL vs. AD, DLB, FTD, *p* < 0.001). No significant differences were shown between patients’ groups (AD vs. DLB vs. FTD) in EVs concentration. Conversely, EVs size was significantly increased in AD, DLB, and FTD samples as compared to CTRL samples (Table 1 and Figure 1C) (*p* < 0.001, one-way ANOVA test with Bonferroni’s post test, CTRL vs. AD, FTD, *p* < 0.001; CTRL vs. DLB, *p* < 0.01). No significant differences were shown between patients’ groups (AD vs. DLB vs. FTD) in EVs size.

NTF analysis revealed that plasma levels of BDNF were not different among the four groups (Table 1); levels of PGRN were reduced only in FTD samples compared to CTRL samples (Table 1) (*p* < 0.01; Kruskal–Wallis test with Dunn’s post test, CTRL vs. FTD, *p* < 0.01). CysC levels were increased in DLB compared to CTRL (Table 1) (*p* < 0.05; Kruskal–Wallis test with Dunn’s post test, CTRL vs. DLB, *p* < 0.05). Plasma GDNF was not detectable in all samples.

**FIGURE 2:**
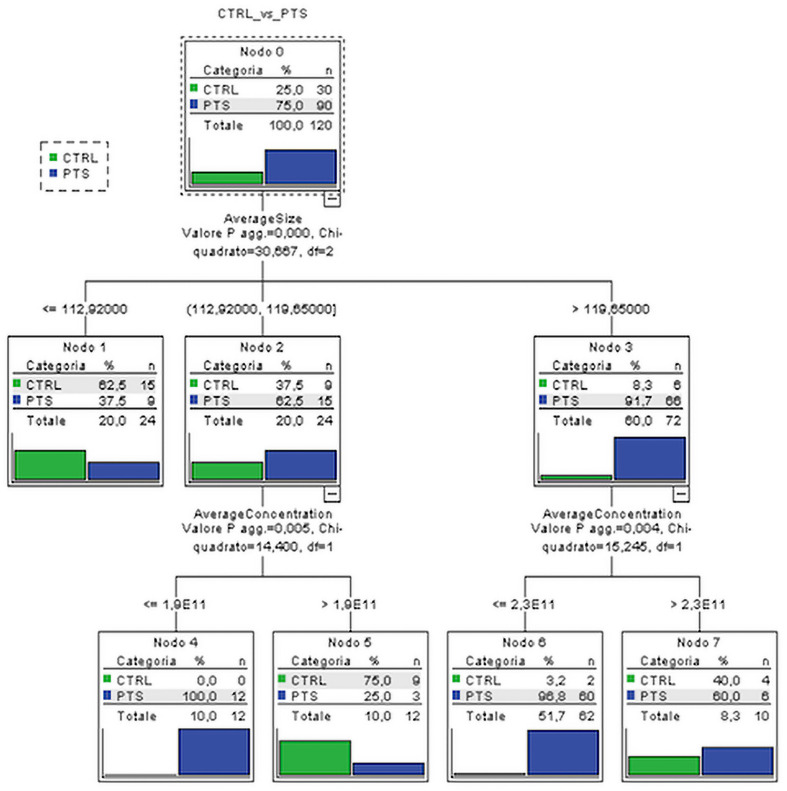
Classification tree. Subjects are classified on the basis of the most predictive variables, EVs size and concentration, among all the ones which resulted to be significantly associated with subject groups. CTRL, controls; PTS, patients; AverageSize, EVs size; AverageConcentration, EVs/ml.

Considering the socio-demographic, EVs, and NTF variables significantly associated with the diagnostic groups, two classification trees were performed respectively on (i) four groups of outcome variable (CTRL, AD, FTD, DLB) and on (ii) dichotomized group variables (CTRL vs. all patients—PTS) in order to detect the predictors that best classify subjects into CTRL or patients. The best classification was obtained using dichotomized group variables in which the average EVs size and average EVs concentration resulted to be the best predictors, i.e., the variables able to better classify the subject into CTRL or PTS. Although we considered all the socio-demographic and NTF in the classification tree, the best discriminant performance was found for EVs variables. Specifically, the EVs size alone (with values larger than 119.7 nm) could classify the majority of dementia patients from controls (91.7% vs. 8.3%). Moreover, among the subjects, the ones with an EVs concentration lower than 2.3 × 10^11^ particles/ml were classified as PTS with very high percentage (96.8%, Figure 2). The inclusion of all significant variables in CT leads to results that were adjusted, and thus robust, for possible confounding effects of the socio-demographic and clinical variables.

**FIGURE 3:**
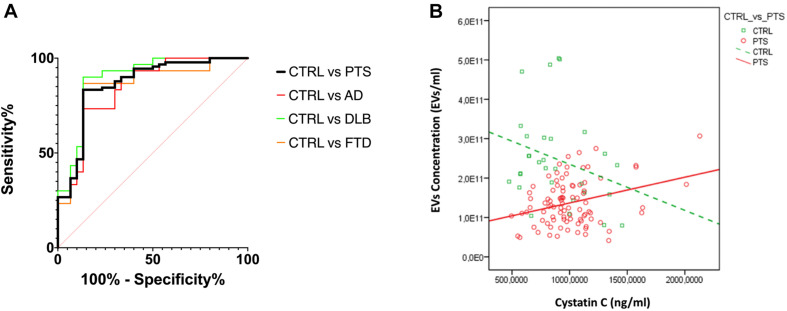
**(A)** ROC curves for EVs concentration/size. The ratio of EVs concentration/size was used to evaluate the discrimination of PTS from CTRL; AUC CTRL vs. PTS 0.86 (black); CTRL vs. AD 0.84 (red); CTRL vs. DLB 0.89 (green); CTRL vs. FTD 0.84 (orange); AUC comparison with DeLong test, *p* > 0.436. **(B)** CysC levels and EVs concentration are negatively/positively associated with CTRL/PTS, respectively (std beta = –0.28; std beta = 0.31 respectively). CTRL, controls (green); PTS, patients (red).

To estimate the diagnostic performance of these two EVs parameters (concentration and size), we performed a ROC analysis to measure the ability of the EVs concentration/size ratio to distinguish dementia patients from CTRL (Figure 3A): an AUC of 0.86 was calculated for the whole PTS group and a sensitivity of 83.3% and specificity of 86.7% with a cut off point of 1.49 × 10^9^. As expected, ROC curves for AD, DLB, and FTD had a similar AUC (0.84, 0.89, 0.85, respectively, with DeLong test *p*-values for AUC comparisons all larger than 0.436).

Finally, in order to evaluate whether EVs size and concentration might be influenced by the NTF, we performed a linear regression analysis between these variables by adding the interaction with dichotomized (CTRL vs. PTS) groups. We found that only levels of CysC were associated with EVs concentration and in a different way between groups (group × CysC effect: *p* = 0.007) (Figure 3B): in CTRL, CysC levels were negatively associated with EVs concentration (standardized beta coefficient = −0.28), while in PTS the association between these two parameters was positive (standardized beta coefficient = 0.31). A positive association was found for all three dementias (data not shown). No significant correlations were found between EVs size and concentration and disease duration (years), as well as disease severity (MMSE) among patients: Spearman’s rho < 0.191 for all the four correlations (*p* > 0.096 for all correlations, data not shown).

## Discussion

Extracellular vesicles represent a new concept in the biomarker field, serving as transfer vehicles between cells of membrane and cytosolic proteins, lipids, DNA, and RNA with a wide range of regulatory functions (Raposo and Stoorvogel, 2013).

In this study, we provide evidence that an alteration in EVs release is common across the three most common neurodegenerative dementias, AD, DLB, and FTD and that plasma EVs dosage and size characterization might be a promising marker for dementias. The differential diagnosis between dementias was not supported by biomarkers for all patients: this could be a limitation of the study (reducing the likelihood of finding differences).

Specifically, we analyzed plasma EVs in three groups of patients affected by AD, DLB, and FTD, and we found a significant reduction in EVs concentration in all groups: −40%, −50%, and −45%, respectively. EVs size was also altered across dementias: in all patients’ groups, we detected a larger plasma EVs size, and specifically +18% in AD, +12% in DLB, and +15% in FTD. We then investigated whether the loss of a neurotrophic factor is also a common pathogenic mechanism among FTD, DLB, and AD and if levels of neurotrophic factor might affect EVs release. To this aim, we chose four candidates that were previously demonstrated to be altered in dementias: BDNF, PGRN, CysC, and GDNF. Plasma levels of PGRN and CysC were partially altered, and specifically PGRN was reduced in FTD while CysC was increased in DLB; however, taking together all variables significantly associated with the diagnostic groups, including PGRN and CysC, only the two parameters related to EVs, i.e., EVs concentration and EVs size, were able to distinguish patients from controls. The diagnostic performance of these two EVs parameters together (concentration/size) was high, with a sensitivity of 83.3% and a specificity of 86.7% (with a cut off point of 1.49 × 10^9^ of the EVs concentration/size ratio). As expected, this ratio could accurately distinguish patients from controls but was not able to differentiate different forms of dementias. A reduction of circulating EVs in dementia is in accordance with our previous studies on FTD and AD cellular models, demonstrating a loss of exosome/exosome-associated CysC in the genetic forms of these diseases (Ghidoni et al., 2011; Ghidoni et al., 2018). These data suggest that an alteration of EVs release is also present in sporadic dementia and it is a common alteration in the three main dementia forms. Of note, plasma EVs alteration is not associated with disease duration and dementia severity.

To the best of our knowledge, this is the first study describing an alteration in the number and size of blood-derived EVs across dementias. Other studies in blood-derived EVs demonstrated an alteration of their cargo in AD, and specifically increased levels of Aβ and tau/p-tau181 (Fiandaca et al., 2015), a dysregulation of a panel of miRNA (Serpente et al., 2020), and a reduction of survival factors (Goetzl et al., 2015, 2016, 2018). In CSF, EVs were increased in subjects with mild cognitive impairment and in AD with respect to controls (Agosta et al., 2014; Joshi et al., 2014). Furthermore, in CSF EVs the percentage of p-tau181, relative to t-tau, appears increased starting from the early stages of AD (Saman et al., 2012). We further highlighted the potential of circulating EVs as a biomarker, and specifically our data suggest an alteration in EVs production (as measured by different circulating EVs concentrations and size) that is occurring in all neurodegenerative dementias, independently from the cargo. Of note, among the candidate NTF only CysC levels were associated with EVs concentration. In dementia patients, CysC was positively associated with EVs, suggesting that this neuroprotective factor, as well as an anti-amyloidogenic protein, might affect EVs release. In line with this observation, we demonstrated *in vitro* (primary neurons from CysC knockout mice) and *in vivo* (CysC transgenic mice) that CysC enhances brain-EVs secretion, resulting in a protective effect (Pérez-González et al., 2019).

In conclusion, our study suggests that an alteration in intercellular communication mediated by EVs might be a common molecular pathway underlying neurodegenerative diseases leading to dementia. CysC might be one of the determinants affecting EVs release, representing a potential therapeutic tool. The identification of shared disease mechanisms is of pivotal importance to identify novel potential therapeutic targets and to develop treatments to delay, slow, or block disease progression. To this aim, further studies investigating plasma EVs as early biomarkers of dementia are required.

## Data Availability Statement

The datasets of raw data generated for this study can be found in the Mendeley Data Repository (doi: 10.17632/2pyfv3tjp9.1).

## Ethics Statement

The studies involving human participants were reviewed and approved by the Comitato Etico IRCCS San Giovanni di Dio Fatebenefratelli Brescia. The patients/participants provided their written informed consent to participate in this study.

## Author Contributions

AL, RN, SB, and CS: data generation. AL, CF, LB, and RG: data analysis. RG: supervision and funding acquisition. GB and GF: clinical samples and phenotyping. AL and LB: original draft preparation for the manuscript. All authors contributed to manuscript revision, read, and approved the submitted version.

## Conflict of Interest

The authors declare that the research was conducted in the absence of any commercial or financial relationships that could be construed as a potential conflict of interest.
